# 

**Published:** 1900-02

**Authors:** 


TABLE OF CONTENTS ON LAST ADVERTISING PAGE.

VOL. XV. FEBRUARY, 1900. No. 8.

424
LIBRARY
S. G. O.

### TEXAS Medical Journal.

SUBSCRIPTION, $1.00 A YEAR, IN ADVANCE.

SINGLE COPIES, 10 CENTS.

PUBLISHED AT AUSTIN, TEXAS, BY
F. E. DANIEL, M. D., & S. E. HUDSON, M. D.,
PROPRIETORS.

Eastern Representative: John Guy Monihan, St. Paul Building, 220 Broadway, New York City.

Entered at the Postoffice at Austin, Texas, as Second-class Mail Matter.

